# Can Antibodies Protect Us Against Cardiovascular Disease?

**DOI:** 10.1016/j.ebiom.2016.06.039

**Published:** 2016-06-28

**Authors:** Jan Nilsson

**Affiliations:** Department of Clinical Sciences, Jan Waldenströms gata 35, 20502 Malmö, Sweden

Antibodies represent one the most effective tools to fight invading pathogens used by the immune system. By binding to specific epitopes on virus and bacteria they can neutralize toxins, activate complement and enhance phagocytic clearance. Some antibodies also bind self-antigens helping to clear away apoptotic cells and other debris providing important house-keeping functions. However, the generation of these protective autoantibodies presents a considerable challenge to the immune system because expression of antibodies that react with the wrong self-antigens may cause autoimmune disease. The possible role of antibodies in cardiovascular disease has attracted considerable attention and evidence for both protective and pathogenic roles has been obtained (Tsiantoulas et al., 2014). In the present issue of *EBioMedicine,* Khamis and co-workers report that subjects that go on to suffer from coronary heart disease (CHD, including myocardial infarction, death from coronary heart disease and coronary revascularization) are characterized not only traditional risk factors such as smoking, hypertension and hypercholesterolemia but also by lower levels of total IgG and to some extent also lower levels of IgM (Khamis et al., 2016). Remarkably, those with IgG in the highest tertile had an almost 60% lower risk of CHD than those in the lowest IgG tertile. The association between low IgG levels and CHD remained when controlling for the variables used in the Framingham risk score and inclusion of IgG improved risk prediction and decreased misclassification into low and high risk individuals. The findings could potentially be of considerable clinical importance and raise two interesting questions. First, should analyses of total plasma Ig be used clinically as a biomarker to improve prediction of cardiovascular risk? This is an attractive possibility because the assay for total Ig is simple, robust and relatively cheap. However, as pointed out by the authors these findings first need to be confirmed in other and larger cohorts. This is particularly important since a previous study on dyslipidemic subjects reported that high levels of total IgG was associated with a higher incidence of CHD (Kovanen et al., 1998). It is of course possible that total Ig is a marker higher risk in subjects with dyslipidemia and a marker of lower risk in subjects with hypertension but it is difficult to see a pathophysiological rational for this. Hence, more studies are needed before the clinical value of total Ig measurements in cardiovascular risk prediction can be truly assessed. The second and much more complex question is whether a low level of total Ig is a cardiovascular risk factor, e.g. Do Ig have a protective function in the cardiovascular system? The possible role of B cells in atherosclerosis has been extensively studied in experimental models but with inconsistent results (Tsiantoulas et al., 2014). Most evidence suggests that B1 cells, which produce germline-encoded natural IgM binding to phospholipid epitopes on microorganisms and apoptotic cells, have a protective effect (Binder et al., 2005). This notion is also supported by findings of accelerated atherosclerosis in mice deficient for IgM (Lewis et al., 2009) and reduced atherosclerosis following treatment with natural antibodies (Faria-Neto et al., 2006). In contrast, most experimental evidence support a pro-atherogenic role of B2 cells but it remains to be fully understood whether this effect is dependent of IgG secretion or a cross-talk with pro-atherogenic Th1 cells (Tsiantoulas et al., 2014). Treatment with high doses of polyclonal IgG reduces atherosclerosis in mice (Nicoletti et al., 1998) but it is questionable if IgG at the concentrations normally found in humans can have this effect. There are also reports of athero-protective effects of specific IgG in mouse models of atherosclerosis (Schiopu et al., 2007). These IgG were generated against certain aldehyde-modified peptide sequences in apolipoprotein B-100 and reduce inflammation through binding to the inhibitory Fc-gamma receptor IIB (Li et al., 2013). However, treatment with these antibodies failed to reduce carotid plaque inflammation in patients with stable cardiovascular disease in a randomized clinical trial (Lehrer-Graiwer et al., 2015). It remains to be clarified whether the negative outcome of this study was due to a lack of athero-protective effects of the antibody in humans or if the level of plaque inflammation in these stable patients was too low for the antibody to have a detectable effect. Notably, Khamis and co-workers found a stronger association between total IgG and CHD than between IgG against aldehyde-modified LDL and CHD. Since IgG against aldehyde-modified LDL only represent one subclass of anti-oxidized LDL antibodies this does not exclude that other types of oxidized LDL antibodies may explain the association between total IgG and cardiovascular risk. However, it is also possible that the latter association is dependent on entirely different mechanisms such as an improved protection against infections.

The findings reported by Khamis and co-workers will stimulate others to further explore the possible role of antibodies in cardiovascular disease. If adding measurements of total IgG can be confirmed to improve cardiovascular risk prediction by currently used scores this would represent a significant clinical breakthrough. It would also strengthen the case for the immune system as a novel target for prevention and treatment of cardiovascular disease.

## Disclosure

This work was partly funded by grants from the Swedish Heart and Lung Foundation (20140078). Jan Nilsson is signed as co-inventor on patents for Immuno-modulation of atherosclerosis owned by CardioVax, Los Angeles, USA.
